# Neurotoxic Effect of Fipronil in Male Wistar Rats: Ameliorative Effect of L-Arginine and L-Carnitine

**DOI:** 10.3390/biology10070682

**Published:** 2021-07-19

**Authors:** Yasmina K. Mahmoud, Ahmed A. Ali, Heba M. A. Abdelrazek, Tahany Saleh Aldayel, Mohamed M. Abdel-Daim, Menna Allah I. El-Menyawy

**Affiliations:** 1 Department of Biochemistry, Faculty of Veterinary Medicine, Suez Canal University, Ismailia 41522, Egypt; yasmina_aziz@vet.suez.edu.eg; 2 Hygiene, Zoonosis and Animal Behavior Department, Faculty of Veterinary Medicine, Suez Canal University, Ismailia 41522, Egypt; ahmedabdelatif@vet.suez.edu.eg; 3 Department of Physiology, Faculty of Veterinary Medicine, Suez Canal University, Ismailia 41522, Egypt; 4 Nutrition and Food Science, Department of Physical Sport Sciences, Princess Nourah Bint Abdulrahman University, Riyadh 11671, Saudi Arabia; TSALdayel@pnu.edu.sa; 5 Department of Pharmacology, Faculty of Veterinary Medicine, Suez Canal University, Ismailia 41522, Egypt; abdeldaim.m@vet.suez.edu.eg; 6 Department of Physiology, Faculty of Medicine, Suez Canal University, Ismailia 41522, Egypt; mennaelmenyawi@med.suez.edu.eg

**Keywords:** doublecortin, fipronil, Iba-1, L-arginine, L-carnitine, oxidative stress, S-2A receptors

## Abstract

**Simple Summary:**

Insecticides are widely used in agricultural and household environments. They induce wide range of deleterious effects. Fipronil is one of the most widely used phenylpyrazoles insecticides. The neurotoxic effect of such insecticide was tested in the present study with special emphasis on cognitive deficit as well as testing the possible ameliorative impacts of L-arginine and L-carnitine. The study proposed fipronil-induced cognitive deficit as a reflection to oxidative stress and neuro-inflammation. Moreover, L-arginine and L-carnitine exerted ameliorative influence on fipronil induced oxidative stress and neuro-inflammation. Therefore, L-arginine and L-carnitine can be considered as prospective candidates for mitigation of pesticide induced neurotoxicity especially in people with high-risk exposure to pesticide.

**Abstract:**

The ameliorative effect of L-arginine (LA) and L-carnitine (LC) against fipronil (FPN)-induced neurotoxicity was explored. In this case, 36 adult male rats were randomly divided into six groups: group I received distilled water, group II received 500 mg/kg LA, group III received 100 mg/kg LC, group IV received 4.85 mg/kg FPN, group V received 4.85 mg/kg FPN and 500 mg/kg LA and group VI received 4.85 mg/kg FPN and 100 mg/kg LC for 6 weeks. Cognitive performance was assessed using Barnes maze (BM). Serum corticosterone, brain total antioxidant capacity (TAC), malondialdehyde (MDA) and dopamine were measured. Histopathology and immunohistochemistry of ionized calcium-binding adaptor (Iba-1), doublecortin (DCX) and serotonin (S-2A) receptors were performed. Fipronil induced noticeable deterioration in spatial learning and memory performance. In addition, FPN significantly (*p* < 0.05) diminished brain antioxidant defense system and dopamine coincide with elevated serum corticosterone level. Histopathological examination revealed degenerative and necrotic changes. Furthermore, Iba-1 and DCX were significantly expressed in cortex and hippocampus whereas S-2A receptors were significantly lowered in FPN group. However, administration of LA or LC alleviated FPN-induced deteriorations. In conclusion, LA and LC could be prospective candidates for mitigation of FPN-induced neurotoxicity via their antioxidant, anti-inflammatory and neuropotentiating effects.

## 1. Introduction

Frequent exposure to environmental toxicants, comprising pesticides, is a definite cause of impairment of neurological functions [1,2]. Pesticides are used in public health to kill vectors of disease; such as pests in agriculture [3]. They are potentially toxic to other organisms, including humans and need to be used safely and disposed of properly. Fipronil (FPN) [5-a m i n o- 3-c y a n o-1-(2,6-d i c h l o r o-4- trifluoromethylphenyl)-4-fluoromethylsulfinyl pyrazole] is a broad-spectrum insecticide that belongs to the phenylpyrazole chemical family and classified as a World Health Organization (WHO) class II moderately hazardous pesticide. It is effective in controlling the insects that are resistant to other insecticides by blocking Gamma aminobutyric acid (GABA) chloride channels in insect’s central nervous system (CNS) [4]. FPN also elicits neurotoxicity in mammals by binding strongly to GABA chloride channels causing hyperexcitability. Furthermore, fipronil sulfone, the main metabolite of FPN in insects and mammals, possesses greater affinity to mammalian GABA receptors than those of insects indicating mischievous influences of FPN catabolic products on non-target organisms [5]. The inhibition of GABA in the prefrontal cortex causes a delay in cognitive tasks and also GABAergic interneurons forming path between entorhinal cortex and hippocampus which constitutes a cornerstone in the spatial and perceptual memory formation [6]. The decrease in GABA release blocks the intra-neural signaling transduction inhibiting presynaptic Ca^2+^ channels. The later causes prevention of neurotransmitter release. Rats treated with FPN showed decreases in hypothalamic, hippocampus and striatum serotonin levels [7]. Moreover, the decrease in GABA plays a fundamental role in neuronal development and synaptic pruning [8].

L-arginine (LA) is a proteinogenic amino acid having important roles in both health and disease [9]. Several human and experimental animal studies had indicated that exogenous LA intake possessed multiple beneficial effects. Its metabolism led to formation of wide variety of biochemically active compounds including glutamate, which is an excitatory neurotransmitter, creatine which is a source of energy, agmatine and polyamines which support neuron functions and proliferation. In addition, nitric oxide (NO) can be produced from LA that promotes optimal cerebral blood flow, consolidates memory processes, facilitates long-term potentiation (LTP) and maintains sleep-wake cycle [10,11]. 

L-carnitine (LC) is an essential nutrient that plays a vital role in energy production and fatty acid metabolism. L-carnitine optimizes cell energy production by transporting long chain fatty acids into the mitochondria for utilization in metabolism through β-oxidation. Moreover, LC protects against neurotoxicity, enhances acetylcholine activity and consequently the cognitive abilities [12]. In this study, we aimed to assess the ameliorative effects of LA and LC against the detrimental neurotoxic effect of FPN.

## 2. Materials and Methods

### 2.1. Animals

In this case, 36 Wistar adult male rats were bought from the Laboratory Animal House, Faculty of Veterinary Medicine, Suez Canal University, Egypt. The weight of the rats ranged from 210 to 230 g. Before the commencement of the study, the rats were left for 1 week as a routine program to be adapted to the surrounding environment. Three rats per cage were kept in a room with sawdust-covered floor and controlled temperature (25 ± 2 °C). The rats were permitted for unrestricted admission to standard diet and water. The procedures of this experiment were carried out under the approval (No. 2021012) and the guidelines of the committee of Scientific Research and Biological Ethics for animals used in laboratory experiments in the Faculty of Veterinary Medicine, Suez Canal University, Egypt.

### 2.2. Fipronil, L-Arginine and L-Carnitine

Fipronil (Coash SC 20%), a preparation from Star Chem. Company (Wellford, SC, USA) and manufactured by Zhejiang Yongnong Chem. Co. (Shaoxing, China), was used in the current experiment. L-arginine was obtained from NOW Foods Company (Bloomingdale, IL, USA). L-carnitine was acquired from Mepaco Co. (Cairo, Egypt). 

### 2.3. Experimental Design

The rats were assigned randomly into six groups, each group had six rats.

Group I, Control rats received only distilled water.
Group II (L-arginine, LA), Rats were gavaged 500 mg/kg LA (25% *w*/*v* in distilled water) [13].
Group III (L-carnitine, LC), Rats were gavaged 100 mg/kg LC [14].
Group IV (Fipronil, FPN), Rats were treated with 4.85 mg/kg FPN (1/20 of FPN LD50). The dosage was chosen according to the available publications regarding oral LD50 of fipronil for rats [15].
Group V (FPN + LA), Rats were gavaged 500 mg/kg LA (25% *w*/*v* in distilled water) and 4.85 mg/kg FPN (1/20 of FPN LD50), one hour apart.
Group VI (FPN + LC), Rats were gavaged LC 100 mg/kg and 4.85 mg/kg FPN (1/20 of FPN LD50), one hour apart.

The doses were given via gastric tube daily for consecutive 6 weeks. 

### 2.4. Body and Brain Weights

The body weights were checked at the beginning and at the end of the study. At the end of experimental period, the animals were euthanized and brains were weighed.

### 2.5. Barnes Maze (BM)

The behavioral test and the room preparation were carried out as described by Barnes [16]. Three main procedures were carried out. Habituation: animals were accommodated to human touch prior to behavioral experimentation by moving each animal from the box and frequent handling and touching were applied for 5 min then the animals were returned to the box again. Rats were habituated to BM spatial memory and learning paradigm one day before starting acquisition phase to decrease anxiety which could alter rat behavior. Habituation was performed by allowing the rats to freely explore the maze for 1 min followed by gentle guiding the rats to the escape hole for about 2 min. The surrounding visual cues were placed as 3 card carton chips (circle, triangle and star). These cues were remained unaltered during habituation, acquisition and probe trial phases. Acquisition phase (to assess spatial learning or acquisition): each acquisition trial started by placing the rat in the middle of the platform in that was covered by dark cylindrical start box, which is then lifted after 15 s, thus allowing the rat to freely explore the platform and locate the target hole. This was repeated for all rats for 4 consecutive training days. Cleaning the platform of the maze was accomplished by 70% ethyl alcohol to remove smelling cues. Probe trial (to assess short term spatial memory retention): on day 5, the escape box was removed and all the cues remained in the same place. The rats were tested for remembering what had been previously learned. Acquisition in the training phase was typically assessed by time latency for each rat to reach the targeted hole.

### 2.6. Blood and Samples Collection 

Blood Samples Were Drawn from retro-orbital venous plexus under effect of light tetrahydrofuran inhalation anesthesia into plain tubes at 7:00 am. Sera were separated and stored at −80 °C until corticosterone and other biochemical markers were estimated. Brains were weighed and divided medially into two equal halves. One half was immersed in 10% formalin for histopathology and the other half was subjected to further homogenization. 

### 2.7. Brain Homogenate

One half was washed with cold phosphate buffer saline (PBS) in order to eliminate blood contamination then dried by filter paper. The cerebral cortex and hippocampus were dissected. Homogenization of cerebral cortex and hippocampus was performed, separately, in cold phosphate buffer (PH = 7.4) then centrifuged at 4000 rpm in a cold centrifuge. The supernatants were kept at –80 °C until malondialdehyde (MDA), total antioxidant capacity (TAC), dopamine analysis. The other brain half was immersed in 10% neutral buffered formalin. 

### 2.8. Total Anti-Oxidant Capacity (TAC) and Malondialdehyde (MDA)

The cerebral cortex and hippocampal homogenate MDA contents, as an indicator of lipid peroxidation and TAC were assayed using commercial ELISA kit (Cat No., MBS268427, MyBioSource Co., San Diego, CA, USA) and (Cat No., MBS733414, MyBioSource Co., San Diego, CA, USA), respectively. All steps were carried out according to the manufacturer’s protocol. 

### 2.9. Dopamine and Corticosterone Levels

Cerebral cortex homogenate dopamine (CSB-E08660r, Cusabio Co., Wuhan, China) and serum corticosterone (CSB-E07014r, Cusabio Co., Wuhan, China) levels were assayed using specific ELISA kits. Procedures were followed according to manufacturer’s instruction.

### 2.10. Histopathology

Formalin-fixed halves of brain tissues were desiccated in gradated alcohols and removed by xylene thereafter submerged in paraffin. Histological sections were sliced into 4–5 µm and stained by Haematoxylin and Eosin stain (H&E) according to Culling et al. [17]. 

### 2.11. Immunohistochemistry (IHC)

Paraffin-embedded brains were sliced into 5 µm sections on positively charged slides for serotonin (S-2A) receptors, ionized calcium-binding adaptor (Iba-1) and doublecortin (DCX) immunohistochemistry. The following primary antibodies; (# sc-32538, Santa Cruz Biotechnology, Dallas, TX, USA) in concentration 1:100, (# sc-32725, Santa Cruz Biotechnology, Dallas, TX, USA) in concentration 200 µg/mL and (# sc-271390, Santa Cruz Biotechnology, Dallas, TX, USA) in concentration 1:400, respectively, were used. Protocol for IHC was performed as described by Schacht and Kern [18] for S-2A receptors, Farrag et al. [19] for Iba-1 and Sirerol-Piquer et al. [20] for DCX. The quantitative analysis of immunoreactive parts percentage (IRP%) was implemented using Image J software. Seven random microscopic fields per slide were subjected to analysis after subtraction of light background.

### 2.12. Statistical Analysis

Results were assessed for normality and data sets were found to follow the normal distribution. Data were represented as mean ± standard error. SPSS 20 software (SPSS Inc., Chicago, IL, USA) was used to perform the analysis. The differences between groups were analyzed by one-way analysis of variance (ANOVA) followed by Duncan’s multiple range post hoc test. *p* < 0.05 refers to significant differences between groups.

## 3. Results

### 3.1. Body and Brain Weights 

The final body weights exhibited significant (*p* < 0.05) reduction in FPN treated group than the control. Administration of LA or LC with FPN resulted in significant (*p* < 0.05) increase in final body weights than FPN treated group. However, initial body weights exhibited non-significant differences between experimental groups. The brain weight of all experimental groups showed non-significant variation (Table 1). 

### 3.2. Barnes Maze

**Figure 1:**
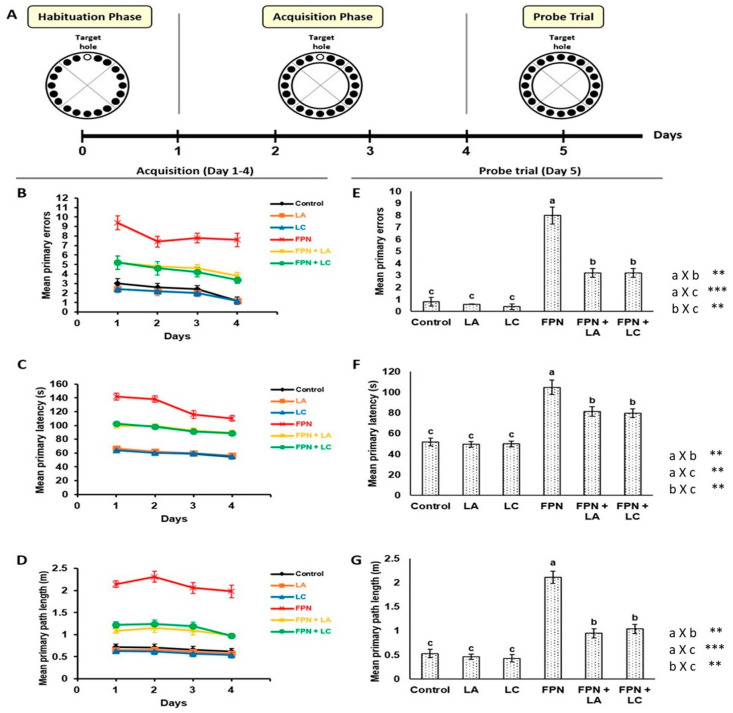
Assessment of spatial learning and spatial memory performance by Barnes maze (BM). (**A**): Diagram illustrated phases of BM paradigm: Habituation, acquisition and probe trial. Effect of L-arginine (LA) and L-carnitine (LC) on BM tasks in rats exposed daily to fipronil (FPN) for 6 weeks during acquisition phase, (**B**): Mean primary errors to locate escape hole, (**C**): Mean primary latency (s) to find escape chamber, (**D**): Mean primary path length (m) to reach target hole, and probe trial, (**E**): Mean primary errors to find target hole, (**F**): Mean primary latency (s) to reach escape hole, (**G**): Mean primary path length (m) to locate target hole in rats exposed daily to FPN. Symbols **, *** indicates significant *p* value < 0.01 and 0.001, respectively.

Cognitive performances of rats were evaluated by BM behavioral test that is used to examine visuo-spatial learning and memory (Figure 1A–G). Data represented during acquisition phase showed decreased primary latency, path length and number of primary errors across training days that declared that all rats had learned the task.

#### 3.2.1. Acquisition Phase

Results showed significant (*p* < 0.05) increase in travelled distance and time needed to locate the target box with elevated number of committed errors in FPN-treated rats when compared to the control. These data demonstrated that prolonged FPN exposure could greatly alter spatial learning. On the contrary, LA and LC supplementations significantly (*p* < 0.05) reduced primary distance and latency to identify the target hole simultaneously with lesser number of working errors (Figure 1B–D).

#### 3.2.2. Probe Trial

Fipronil treatment resulted in marked (*p* < 0.05) elevations in primary latency, number of working errors and path length to reach target hole whereas, FPN + LA or LC treated rats required (*p* < 0.05) less time, spent less distance and performed lesser errors to achieve the task (Figure 1E–G).

### 3.3. Total Anti-Oxidant Capacity and Malondialdehyde Levels

The overall mean values of cortical and hippocampal TAC and MDA contents were shown in Table 2. The TAC contents significantly (*p* < 0.05) decreased in FPN-treated rats compared to controls. Upon comparing FPN + LA and FPN + LC treated groups to FPN-treated group, the TAC values were found to be significantly (*p* < 0.05) increased. However, concentrations of MDA significantly (*p* < 0.05) elevated in rats exposed to FPN compared to the control group. Concentrations of MDA significantly (*p* < 0.05) suppressed in rats of both FPN + LA and FPN + LC groups compared to FPN group.

### 3.4. Dopamine and Corticosterone Levels

Rats receiving FPN revealed significantly (*p* < 0.05) higher serum corticosterone levels when compared to that in the control group. Meanwhile, FPN + LA and FPN + LC rats exhibited marked (*p* < 0.05) improvement in corticosterone level when compared to animals exposed to FPN (Table 3). Brain dopamine concentration was diminished significantly (*p* < 0.05) in FPN-treated group as compared to the control group. On the other hand, oral administration of LA or LC with FPN induced a significant (*p* < 0.05) increase in dopamine concentration (Table 3).

### 3.5. Histopathology

**Figure 2:**
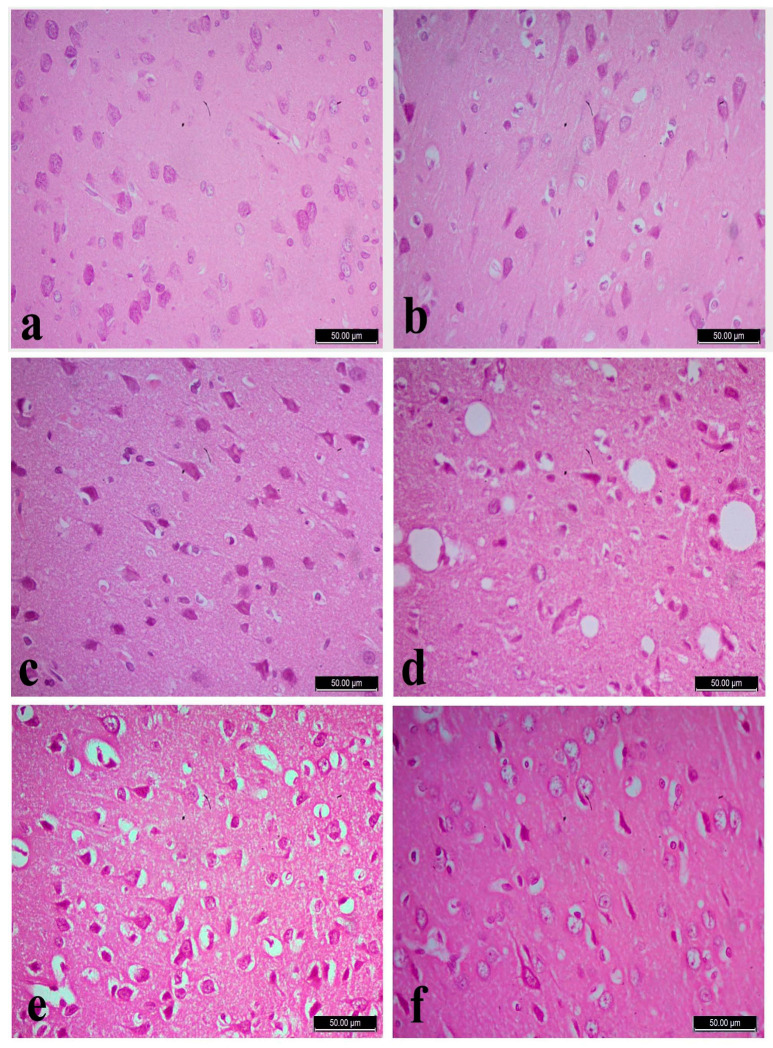
Cerebral cortex of control (**a**), L-arginine **(b**), L-carnitine (**c**) showed normal neurons and glia cells. Fipronil (FPN)-treated cortex (**d**) showed neurons with necrotic changes in the form of hyperchromatic nuclei (arrow heads), chromatolysis, degenerated and vacuolated neurocytes (arrows). Pronounced improvement of neurocytes and most of them appeared normal with large vesicular nuclei in both FPN + L-arginine and FPN + L-carnitine-treated groups (**e**,**f**). Stain: Hematoxylin and Eosin (H&E), magnification 400×.

The cerebral cortex of the normal control groups showed normal neurons and glia cells among all its six layers (Figure 2a–c). Whereas cortex of the FPN-treated rats showed degenerated and vacuolated neurocytes with necrotic changes in the form of pyknotic and hyperchromatic nuclei (Figure 2d). The cerebral cortex of FPN + LC and FPN + LA -treated rats showed healthy neurocytes resembled the control groups, with central large vesicular nuclei. Pronounced improvement in neurocytes and most of them appeared normal with large vesicular nuclei was observed (Figure 2e,f).

**Figure 3:**
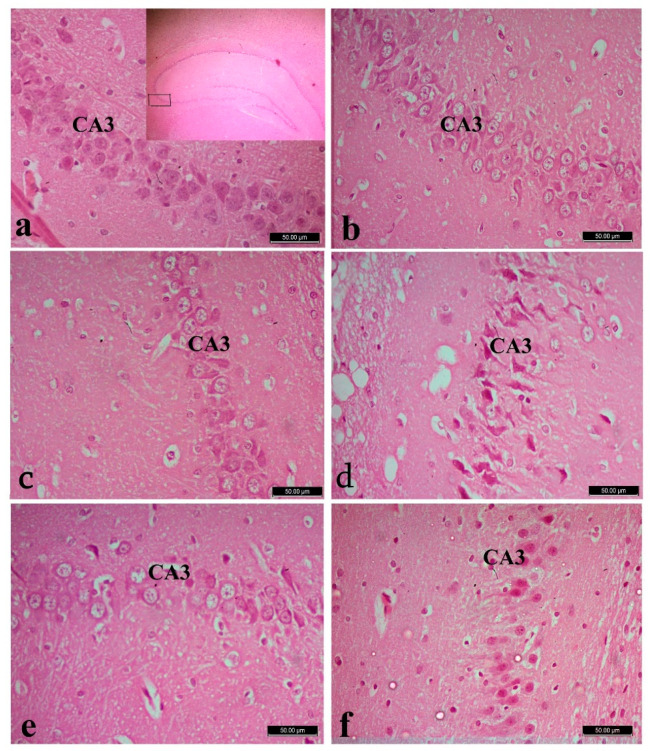
Hippocampus of control (**a**) with right pan window showed the area of CA3 in hippocampus, L-arginine (**b**), L-carnitine (**c**) and fipronil (FPN)-treated rats (**d**). FPN-treated rat showed decreased thickness and degeneration of pyramidal cell layer in the CA3 region, with dystrophic changes in the form of shrunken hyperchromatic, with irregular distribution and degenerated neurocytes. Improvement is observed in FPN + L-arginine and FPN + L-carnitine-treated groups (**e**,**f**). Stain: Hematoxylin and Eosin (H&E), magnification 400×.

The hippocampal region of control, LA and LC groups has three distinguished components: the hippocampus proper (Ammon’s horn), the dentate gyrus and subicular cortex. The hippocampus proper consisted of cornu ammonis (CA). Cornu Ammonis (CA) was differentiated into CA1, CA2, CA3 and CA4. In cornu ammonis, the pyramidal cells in the pyramidal cell layer of CA3 were triangular with processes, large perikarya, basophilic cytoplasm, large vesicular nuclei and prominent nucleoli (Figure 3a–c). In FPN-treated groups, CA areas of the pyramidal cell layer showed many necrotic changes with shrunken perikarya among few normal pyramidal cells with vesicular nuclei (Figure 3d). The FPN + LA and FPN + LC -treated groups had apparently normal to healthy pyramidal cells (Figure 3e,f).

**Figure 4:**
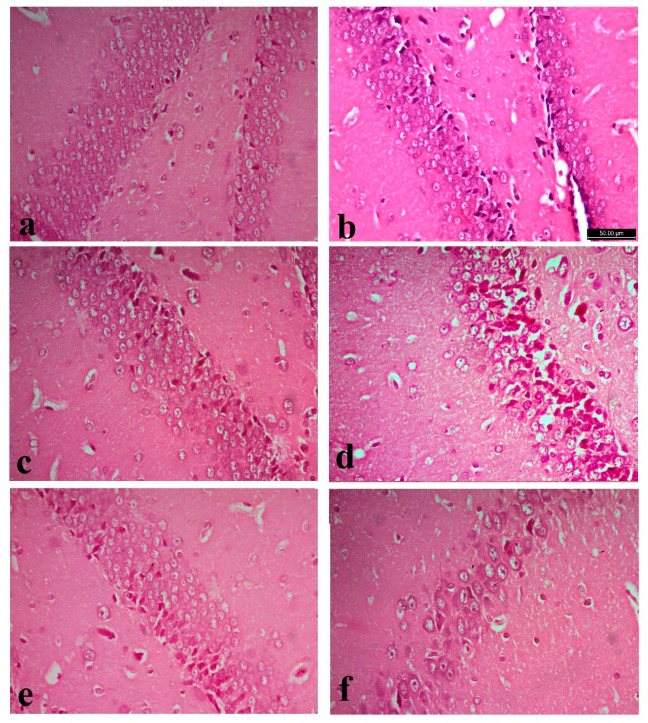
Dentate gyrus of control (**a**), L-arginine (**b**), L-carnitine (**c**) and fipronil (FPN)-treated rats (**d**). FPN-treated rat (**d**) showed shrunken, darkly stained granule cells with cytoplasmic vacuolations among few nearly normal granules cells with large vesicular nuclei. Improvements were observed in FPN + L-arginine and FPN + L-carnitine-treated groups (**e**,**f**). Stain: Hematoxylin and Eosin (H&E), magnification 400×.

The dentate gyrus of control, LA and LC groups consists of three-layer structure: a superficial molecular layer; an intermediate granular cell layer and a deep polymorphic cell layer. The granule cells were small, rounded with large vesicular nuclei and prominent nucleoli tightly packed together in 8 to 10 layers in the stratum granulosum (Figure 4a–c). The dentate gyrus of FPN group showed shrunken, darkly stained granule cells with cytoplasmic vacuolations (Figure 4d). Improvement of granular and subgranular cells were observed in LA and LC treated groups (Figure 4e,f).

### 3.6. Immunohistochemistry

**Figure 5:**
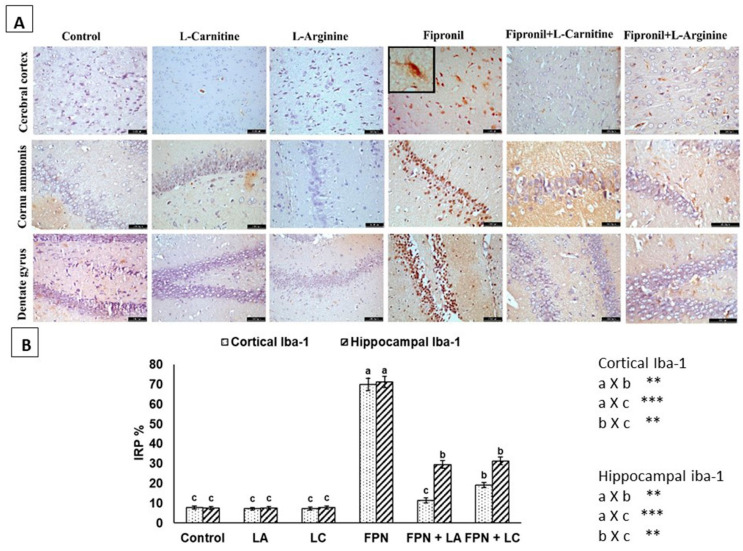
(**A**) Immunohistochemical staining of the cerebral cortex, CA region and dentate gyrus with Iba-1. Negative and non-observed immunostaining were seen in control, L-arginine (LA) and L-carnitine (LC) groups. Fipronil (FPN) group showed positive brownish Iba-1 immunoreactive microglia with numerous fine branching processes nuclei. Reduced immunoreactivity of microglia in FPN + LA and FPN + LC treated groups was seen [Anti-Iba-1 × 400]. (**B**) Immunoreactive parts percentage (IRP%) of Iba-1 protein expressed as mean ± SE. Symbols **, *** indicates significant *p* value < 0.01 and 0.001, respectively.

Figure 5 revealed the immunohistochemistry results of Iba-1. There was marked increase in the Iba-1 staining of the cerebral cortex, CA region and dentate gyrus activated microglia cells and their processes as dark brown stain in FPN-treated group compared to other groups. The activated stained microglia were observed. A reduction in activation of microglia in treated groups was recorded. However, negative and non-observed immunostaining were observed in control, LA and LC groups (Figure 5A). The IRP% showed significant (*p* < 0.05) elevations in cerebral cortex and hippocampus of FPN administered rats than control. There were significant (*p* < 0.05) reductions in Iba-1 IRP% of FPN + LA group and FPN + LC than FPN group (Figure 5B).

**Figure 6:**
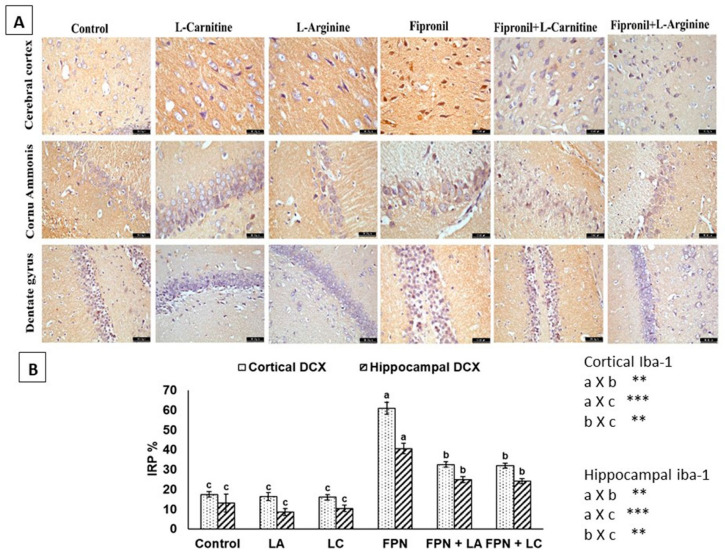
(**A**) Immunohistochemical staining of the cerebral cortex, CA region and dentate gyrus with Iba-1. Weak perinuclear membrane reaction was seen in control, L-arginine (LA) and L-carnitine (LC) groups. Fipronil (FPN) group showed intense positive brownish immunoreactive neurons in the subgranular and granule cell layers. Reduced immunoreactivity of microglia in FPN + LA and FPN + LC treated groups was seen [Anti-DCX × 400]. (**B**) Immunoreactive parts percentage (IRP%) of DCX protein expressed as mean ± SE. Symbols **, *** indicates significant *p* value < 0.01 and 0.001, respectively.

In the cerebral cortex, CA region and dentate gyrus sections stained for DCX, showed weak perinuclear membrane reaction in control, LA and LC groups. A strong positive reaction in FPN-treated group was observed in neurons of both cortex and CA region. Small dark brownish granules in the cytoplasm of the neurons in the subgranular zone and granule cell layer (GCL) of dentate gyrus were also seen. Marked amelioration was observed in FPN + LA and FPN + LC-treated groups (Figure 6A). The IRP% of DCX demonstrated significant (*p* < 0.05) elevations in the cerebral cortex and hippocampus of FPN-treated rats when compared to control. There were significant (*p* < 0.05) reductions in DCX IRP% of FPN + LA group and FPN + LC than FPN group (Figure 6B).

**Figure 7:**
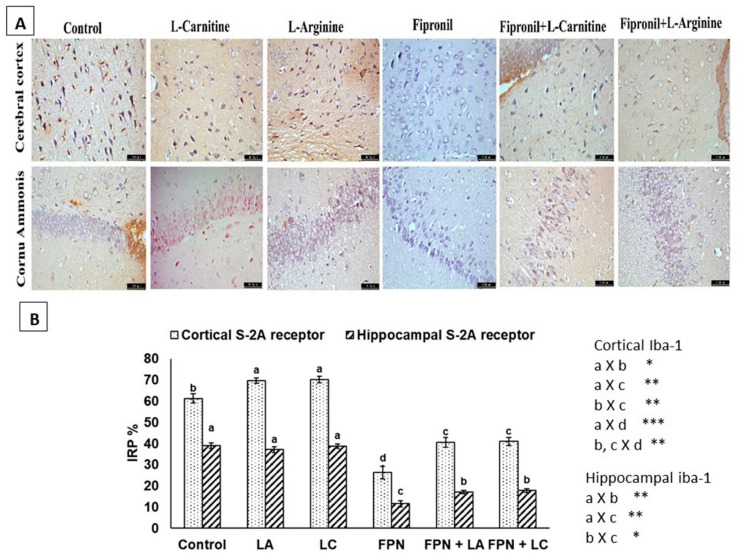
(**A**) Immunohistochemical staining of the cerebral cortex and CA 3 region with serotonin (S-2A) receptors. Positive brownish immunoreactive perikarya in control, L-arginine (LA) and L-carnitine (LC) groups. Negative staining was evident in fipronil (FPN) group. Improvements and promotion of immunostaining were observed in FPN + LA and FPN + LC treated groups [anti-S-2A × 400]. (**B**) Immunoreactive parts percentage (IRP%) of cortical and hippocampal S-2A receptors protein expressed as mean ± SE. Symbols *, **, *** indicates significant *p* value < 0.05, 0.01 and 0.001, respectively.

The control, LA and LC groups revealed immunoreactivity for S-2A receptors in both cerebral cortex and pyramidal cells of CA3 of the hippocampus. Whereas FPN-treated group showed negative immunostaining either in cortex or hippocampus. Positive reactions were observed in both FPN + LA and FPN + LC groups (Figure 7A). The IRP% of S-2A receptors showed significant (*p* < 0.05) increase in cerebral cortex of LA and LC groups than control while hippocampus did not vary more than control. The cerebral cortex and hippocampus of FPN-treated rats exhibited significant (*p* < 0.05) reduction in S-2A IRP% than control. There were significant (*p* < 0.05) improvements in S-2A IRP% of FPN + LA group and FPN + LC than FPN group (Figure 7B).

## 4. Discussion

The widespread use of pesticides in agriculture, domestic and household environments represents a serious threat not only to target species but also to the environment and every living organism exposed to them [21]. Phenylpyrazoles group is one of the most widely used insecticides worldwide due to its great toxicity to the invertebrates, ease of application and systemic nature thus, ensuring their efficient spread to the entire treated area [22]. Implications of pesticides on non-target organisms are considered the cornerstone in pest management programs [23]. Exposure to pesticides, either directly or indirectly, can induce wide variety of toxic effects on non-target organisms including hepatotoxicity, nephrotoxicity, neurotoxicity, hematotoxicity, etc [24,25]. This study was designed to evaluate biochemical and cognitive consequences induced by subchronic exposure to one of the most widely used pesticide, FPN and the possible alleviative effects of LA or LC.

Biochemical, functional and dynamical interactions between hippocampus and prefrontal cortex is critical for cognition, spatial navigation and mnemonic processing [26]. Learning and memory are fundamental cognitive processes involving acquiring, storage and retrieving knowledge or information. They perform crucial functions by enabling the organism to change its behavior in response to variable environmental changes [27]. Prolonged stress due to frequent exposure to pesticides is hypothesized to be associated with physiological, cognitive and behavioral alterations including spatial memory and learning disorders [13]. Barnes maze is a valuable method to assess the impairments in spatial learning and memory in rodents [28]. Data of this study revealed that FPN dramatically influence rat’s cognitive functions and memory. Compared to the control group, FPN-treated rats displayed markedly impaired spatial acquisition and memory retention. They travelled longer path length and required more time to locate escape hole coincided with markedly elevated errors number in both acquisition and probe phases. The increased latency together with greater error numbers may indicate increased rat anxiety. Similar to our findings, Taibi et al. [29], Godinho et al. [6] and Terçariol et al. [30] who reported disruption of cognitive capabilities in rats exposed to FPN upon investigation using several types of spatial mazes such as BM, eight radial arm maze and elevated plus-maze. The demonstrated retrograde neurophysiological impairments in the current experiment were in line with neurocytes vacuolation and degeneration existed in such group. Many studies had associated cellular and structural alterations in the cortex and hippocampus with deteriorated spatial cognitive performance [31,32,33,34]. Moreover, the cognitive and behavioral deficits, as well as hippocampal degeneration observed in FPN group could be attributed to the damage implicated by FPN-induced oxidative stress (OS) on brain structure and function. Our results showed that treatment of rats with FPN altered brain redox balance. This is demonstrated by the significantly elevated levels of MDA with suppressed TAC in brain homogenate. These findings suggest an increase in brain reactive oxygen species (ROS) and development of OS due to the lipophilic nature of FPN and its metabolites that make them likely to easily pass through blood-brain barrier [35]. OS can seriously influence CNS functions via induction of mitochondrial dysfunction, synaptic impairment, interference with neuronal transmission and suppression of neurogenesis [36,37] resulting in impaired memory and learning capabilities. In agreement with our results, many studies had shown that FPN treatment induced generation of ROS [38,39,40].

Redox balance is crucial for sustaining cellular homeostasis [41]. The imbalance between pro-oxidants and anti-oxidants in favor of the former results in the development of OS [42]. Brain biochemical integrity is pivotal for physiological functions of the CNS. Daily administration of LA or LC simultaneously with FPN retrieved memory and cognitive abilities as well as mitigated hippocampal and cortical degeneration. Both LA [43] and LC [44] were thought to possess anti-oxidant and anti-lipid peroxidation effect in brain tissue which is manifested in the current study via the reduced MDA and elevated TAC in FPN-treated rats given LA and LC. Moreover, LA could enhance efficacy of O_2_ utilization [45] and nitric oxide (NO) production. The latter is an important neurotransmitter that plays a marginal role in maintaining normal functions of the CNS and boosts cerebral blood flow, neural communications, intracellular signal transduction and memory [10,46]. The observed ameliorative effects of LC against FPN-induced OS may be due to its iron-chelation property and lipid peroxidation prohibiting action [47,48,49] where iron is known to be essential for cellular generation of the powerful oxidant, hydroxyl radicals, through Fenton reaction [50]. In addition, LC enhances basal anti-oxidant capacity via elevation of reduced glutathione to oxidized glutathione (GSH/GSSG) ratio [51] and activation of anti-oxidant enzymes including superoxide dismutase, catalase, glutathione reductase and glutathione peroxidase [52,53]. L-carntine could diminish lipid peroxidation via transportation of fatty acids to mitochondria for energy production thus, reducing fatty acids available for peroxidation [54]. In agreement with our results, many studies reported the improved learning and accelerated retrieval of maze tasks following LA [55] and LC [56,57] treatments.

The current study revealed that FPN exposure significantly elevated serum corticosterone level. This finding reflects the stress employed on the animals due to prolonged exposure to the pesticide. Whereas, corticosterone, the main stress hormone, is produced by adrenal cortex, in rats [58] following physical or emotional stress. Its secretion is controlled by corticotropin-releasing hormone and adrenocorticotropic hormone in the hypothalamus-pituitary-adrenal (HPA) axis [59]. Disrupted negative feedback of the HPA axis is related to ROS overproduction [60] that results in cognitive impairments, memory loss and behavioral deficits [61]. Antioxidant influences of both LA and LC efficiently ameliorated the HPA stimulated corticosterone production in the herein study.

Subchronic FPN exposure provoked remarkable inflammatory response. This was manifested by marked elevation in Iba-1 IRP% of the activated microglia cells. This finding is consistent with that obtained by Park et al. [62]. Elevation of Iba-1, the most used marker of microglia activation, in FPN-administered rats could indicate over activated microglia cells and neurotoxic effect. Inflammation and OS are closely linked to each other [63]. Thereby, the elevated OS triggered by FPN is incorporated in brain pro-inflammatory conditions and microglia activation. Herein data is of major significance since neurodegeneration and decline in cognitive functions are closely linked to microglia activation and elevated OS [64]. The current data correlates well with the histopathological alterations developed in cortex and hippocampus of rats subjected to the pesticide. Another explanation is that administration of FPN could inhibit GABA receptors that had been incriminated in impairment of cognitive performance [65] where GABA plays a crucial part in various pathophysiological processes including; information integration, information processing, cognitive function-related neural oscillations and modulation of cortical and hippocampal neural circuitry and activity [66]. Moreover, GABAergic signaling employs a mutual influence over neuroinflammatory processes [67].

Data of current study revealed that suppression of microglia-mediated inflammatory response is one of the possible mechanisms underlying the neuroprotective potential of LA and LC. Co-treatment with either LA or LC markedly alleviated hippocampal and cortical microglial activation induced by FPN thus, suggesting mild inflammatory reaction. The anti-inflammatory property possessed by LA and LC could enhance neural integrity and favor cellular recovery following exposure to injury. This explained potentiation of cognitive functions in these groups. The lowered inflammatory response, manifested by reduced Iba-1 IRP%, that was observed in LA and LC treated rats. may be attributed to the improved TAC in these groups causing diminished O_2_^−^ and ONOO production. Both LA and LC had been documented to regulate immune function and inflammatory response [68,69]. They play fundamental roles in restraining OS and destructive inflammatory reaction [69,70] hence, mitigate neural injury. Moreover, both LA and LC could modulate dependent pathways to reduce neuroinflammation. L-arginine may be hypothesized to activate GABA-dependent membrane currents through a NO-independent mechanism therefore it could potentiate GABA synaptic transmission and plasticity [71] as well as abolishing FPN-induced neuroinflammation. In addition, LC could be metabolized by the tricarboxylic acid cycle and incorporated into the carbon skeleton of glutamate, glutamine and GABA [72]. Therefore, it could improve GABA mediated synaptic transmission as well as lessen FPN-induced neuroinflammation. These characteristics may help to elucidate the beneficial role of LA and LC in repressing the pro-inflammatory response triggered by FPN. The mechanism implicated in anti-inflammatory property of LA and LC could impart due to antioxidant effect [52,73,74] that was clearly demonstrated in the present study.

Dysregulation of adult neurogenesis is a common hallmark in various neurological diseases including epilepsy [75], cognitive dysfunction [76], depression, mood disorders [77], Parkinson’s [78] and Alzheimer’s diseases [79]. A growing body of literature has emphasized the adverse effects of pesticides on pre- and postnatal neurogenesis [80,81,82]. Severity could extend to influence the proliferation of neural progenitor cells, change the developmental destiny of newly-born neurons or even reduce the fundamental cognitive processes [83,84]. Upon examining cerebral cortex and hippocampus, the results showed that repeated daily FPN administration for 6 weeks is associated with marked elevation of IRP% of DCX in the examined areas compared to control group. DCX is an important microtubule protein. It is temporary expressed during adult mammalian neurogenesis by immature newly generated neurons while suppressed before neural maturation [85] therefore, considered as a marker of immature neural proliferation. Along with influencing HPA axis during stress [86], both OS and neuroinflammation induced production of large amount of NO which was accused of demyelination and hindering nerve regeneration [87]. Moreover, it has been emphasized that OS and inflammation could inhibit cell growth and could even trigger apoptosis [88,89] via inducing various ROS-sensitive apoptotic pathways and transcription factors such as caspases and mitogen-activated protein kinases (MAPKs) [90]. It has been hypothesized that FPN may drive upregulation of apoptotic P53, caspase-3 and Bax proteins whereas, down-regulation of anti-apoptotic Bcl-2 protein [91]. Hence, the resulted remarked increase in neuron generation might be interpreted as a body compensatory response to neural loss following daily FPN exposure. These come in agreement with the vacuolated and degenerated neurocytes displayed in cortex and hippocampus of this group. Furthermore, Zhang et al. [92] declared that over activation of microglia could alter neurogenesis and urge depressive like-behavior [92]. In addition, stress is regarded one of the important mechanisms that impacts adult neurogenesis. It triggers production of glucocorticoids via stimulation of HPA axis [93]. The released glucocorticoids persuade a profound consequence on adult neurogenesis through disturbing nerve cell proliferation, differentiation and survival [94].

Despite the beneficial role of adult-born hippocampal neurons in cognitive processes, but adult-generated neurons while still being in an immature phase elucidate that they could not yet been synaptically incorporated into the pre-existent brain neuronal circuits. Our findings declared the detrimental effects of FPN on adult neurogenesis and may help elucidation of the cognitive and behavioral disturbances observed in this group. In harmony with our results, Sidiropoulou et al. and Lassiter et al. [95,96] showed that FPN is a powerful disruptor of neural development and differentiation in mammalian cell lines.

The current study displayed a marked reduction in IRP% of DCX in brain of the rats exposed to LA or LC coincided with the pesticide. This strongly supports the possibility that both treatments, via their antioxidant and anti-inflammatory properties, enhance neuron integrity and viability that may be hypothesized to be related to the diminished neural loss and apoptosis. Another coherent prospect could be that LA and LC enhanced neural regeneration and promoted survival as well as maturation of newly formed neurons. This is reflected as a noticeable improvement in neurocytes structure in the inspected brain sections and their great resemblance to the control group. In this respect, LA and LC could be used for improving neural regeneration, survival and maturation.

Our findings indicated that FPN-treated rats exhibited a significant decrease in brain homogenate dopamine concentration coupled with a marked decline in S-2A receptors IRP%. Both are crucial catecholaminergic neurotransmitters playing vital roles in dominating numerous body functions. They regulate body posture, emotion, behavior, cognition and motor functions [97,98] and are highly liable to environmental toxicants [99,100]. Results of the current study are consistent with those of Bharatiya et al. and Anadón et al. [7,101,102]. The observed cognitive impairment following sustained FPN exposure was believed to be a direct consequence of the pesticide hazardous effects on dopamine neurotransmitter and S-2A receptors that resulted in disruption of brain connectivity. The deleterious impact of FPN on dopaminergic and serotonergic neurotransmission systems could be aroused from the neurotoxic insult of FPN on brain antioxidant system and microglia cells. Oxidative stress and neuroinflammation have been indicated to promote one another and eliminate brain defense mechanism thus, evoked degenerative changes in neurons [103,104,105]. Exaggerated inflammation induced by FPN could result in cumulative loss of nigrostriatal dopaminergic neurons [62] and S-2A receptors [106]. Further, suppressed adult neurogenesis and neural maturation decrease the chance of neural substitution which exacerbate the condition [107] and collaborate in exhaustion of dopaminergic and serotonergic neurotransmission. These influences notably evidence the neurotoxic effect of FPN pesticide on non-target organisms.

On the other side, co-administration of LA or LC with FPN markedly elevated dopamine concentration and restored S-2A receptors. The detected marvelous improvement of brain neurochemistry is consistent with the potentiated cognitive capabilities shown in BM tasks performed by these groups. Moreover, compared to the control group, LA and LC administered animals displayed higher cortical S-2A receptors immunoreactivity which could emphasize their positive influence on brain neurons. These results are in coincidence with those of Strasser et al., Volz and Schenk, Lorrain and Hull and Lechin et al. [108,109,110,111] for LA and Lechin et al., Juliet et al. and Hamza et al. [112,113,114] for LC. Arginine, being a member of glutamate family [115] could be metabolized to glutamate, the fundamental excitatory neurotransmitter in the CNS [116]. Furthermore, LA-NO pathway was implicated in the consolidation of neurotransmission. NO-induced cGMP synthesis enhanced synaptic plasticity and neurotransmission efficacy. This results in reinforcement of cognitive behavior and LTP [117,118]. L-carnitine and its isomer, acetyl-L-carnitine (ALC), have been utilized as cerebral bioenergetics to ameliorate mitochondrial function and promote nerve cells activity [119]. Indeed, this effect has been documented by several studies [112,120].

In this context, the exhibited improvement in dopaminergic and serotonergic systems may be attributed to neuropotentiating effects exerted by both LA and LC which is the outcome of multiple integrating disciplines. These comprise retrieving brain oxidants/antioxidant hemostasis, minimizing generalized stress, relieving neuroinflammation, maintaining neural integrity, consolidating cellular energy metabolism, boosting effective neurogenesis and modulating brain neurochemistry. This system altogether forms an outstanding protection against FPN-induced OS and neurodegeneration and reflected as improvement in spatial memory performance and cognitive flexibility.

## 5. Conclusions

In conclusion, the present study demonstrated that exposure to FPN for 6 weeks could seriously affect spatial memory and cognitive functions of male rats. Mechanisms including OS, persistent inflammation, altered adult neurogenesis and disrupted neurotransmission system were involved in neurodegenerative potential of FPN. Both LA and LC supplementations have been found to exert neuroprotective effects against FPN induced neuronal injury. This was achieved by their antioxidant, anti-inflammatory and neuromodulatory potentials that integrated together to boost neuronal functions. The neuropotentiating actions offered by LA and LC highlight their possible use as protective and/or therapeutic agents for cognitive deficits. Further, this study recommends the use of LA or LC as potential pharmacological strategy in people at high risk to pesticide exposure.

## Figures and Tables

**Table 1:** Effects of L-arginine (LA) and L-carnitine (LC) on body and brain weights in fipronil (FPN)-treated male rats.

	Control	LA	LC	FPN	FPN + LA	FPN + LC	*p* Value
Initial body weight (g)	184.00 ^a^ ± 0.85	179.20 ^a^ ± 2.21	179.30 ^a^ ± 1.87	182.70 ^a^ ± 2.48	179.30 ^a^ ± 1.87	182.30 ^a^ ± 2.9	>0.05
Final body weight (g)	271.70 ^ab^ ± 11.74	305.30 ^a^ ± 11.73	290.00 ^a^ ± 13.04	243.50 ^b^ ± 13.04	288.00 ^a^ ± 13.04	294.70 ^a^ ± 13.04	*
Brain weight (g)	4.12 ^a^ ±0.51	4.17 ^a^ ±0.75	4.13 ^a^ ±0.44	4.01 ^a^ ±0.24	4.06 ^a^ ±0.15	4.04 ^a^ ±0.11	>0.05

Data was expressed as mean ± SE. The different superscripts were significantly differed within the same row. * indicates significant *p* value < 0.05.

**Table 2:** Effects of L-arginine (LA) and L-carnitine (LC) on cortical and hippocampal total antioxidant capacity (TAC) and malondialdehyde (MDA) levels in fipronil (FPN)-treated male rats.

		Control	LA	LC	FPN	FPN + LA	FPN + LC	*p* Value
TAC (U/mg)	Cortical	89.48 ^a^ ± 13.16	90.13 ^a^ ± 11.21	88.67 ^a^ ± 9.29	33.56 ^b^ ± 13.73	62.56 ^ab^ ± 23.39	63.30 ^ab^ ± 19.56	Control × FPN ** LA × FPN *** LC × FPN **
	Hippocampal	93.76 ^a^ ± 7.20	96.75 ^a^ ± 6.81	97.55 ^a^ ± 4.49	39.73 ^b^ ± 13.69	52.90 ^b^ ± 7.55	54.47 ^b^ ± 4.59	***
MDA (U/mg)	Cortical	1.17 ^c^ ± 0.18	1.07 ^c^ ± 0.11	1.01 ^c^ ± 0.03	1.80 ^a^ ± 0.08	1.38 ^b^ ± 0.17	1.38 ^b^ ± 0.10	Control × FPN *** LA, LC × FPN *** LA × FPN + LA * LA × FPN + LC * LC × FPN + LA, FPN + LC ** FPN × FPN + LA, FPN + LC **
	Hippocampal	0.97 ^b^ ± 0.09	1.02 ^b^ ± 0.06	1.03 ^b^ ± 0.06	1.81 ^a^ ± 0.09	1.40 ^c^ ± 0.12	1.38 ^c^ ± 0.04	***

Data was expressed as mean ± SE. The different superscripts were significantly differed within the same row. Symbols *, **, *** indicates significant *p* value < 0.05, 0.01 and 0.001, respectively.

**Table 3:** Effect of L-arginine (LA) and L-carnitine (LC) on serum corticosterone and cortical dopamine levels in fipronil (FPN)-treated male rats.

	Control	LA	LC	FPN	FPN + LA	FPN + LC	*p* Value
Corticosterone (pg/mL)	98.83 ^a^ ± 2.51	96.52 ^a^ ± 1.98	101.78 ^a^ ± 2.39	141.31 ^b^ ± 4.41	116.12 ^c^ ± 6.25	112.65 ^ac^ ± 2.79	FPN × control, LA, LC *** FPN × FPN + LA, FPN + LC * FPN + LA × control, LA *
Dopamine (ng/g tissue)	29.39 ^a^ ± 0.87	31.76 ^a^ ± 0.75	32.02 ^a^ ± 0.89	13.54 ^b^ ± 2.12	20.73 ^c^ ± 2.03	21.00 ^c^ ± 1.83	FPN × control, LA, LC *** LA, LC × FPN + LA, FPN + LC *** FPN × FPN + LA, FPN + LC *

Data was expressed as mean ± SE. The different superscripts were significantly differed within the same row. Symbols *, *** indicates significant *p* value < 0.05 and 0.001, respectively.

## Data Availability

The data presented in this study are available in the article [Neurotoxic effect of fipronil in male Wistar rats: Ameliorative effect of L-arginine and L-carnitine].
