# Correction: Mahmoud et al. Neurotoxic Effect of Fipronil in Male Wistar Rats: Ameliorative Effect of L-Arginine and L-Carnitine. *Biology* 2021, *10*, 682

**DOI:** 10.3390/biology12070904

**Published:** 2023-06-25

**Authors:** Yasmina K. Mahmoud, Ahmed A. Ali, Heba M. A. Abdelrazek, Tahany Saleh Aldayel, Mohamed M. Abdel-Daim, Menna Allah I. El-Menyawy

**Affiliations:** 1 Department of Biochemistry, Faculty of Veterinary Medicine, Suez Canal University, Ismailia 41522, Egypt; yasmina_aziz@vet.suez.edu.eg; 2 Hygiene, Zoonosis and Animal Behavior Department, Faculty of Veterinary Medicine, Suez Canal University, Ismailia 41522, Egypt; ahmedabdelatif@vet.suez.edu.eg; 3 Department of Physiology, Faculty of Veterinary Medicine, Suez Canal University, Ismailia 41522, Egypt; 4 Nutrition and Food Science, Department of Physical Sport Sciences, Princess Nourah Bint Abdulrahman University, Riyadh 11671, Saudi Arabia; tsaldayel@pnu.edu.sa; 5 Department of Pharmacology, Faculty of Veterinary Medicine, Suez Canal University, Ismailia 41522, Egypt; abdeldaim.m@vet.suez.edu.eg; 6 Department of Physiology, Faculty of Medicine, Suez Canal University, Ismailia 41522, Egypt; mennaelmenyawi@med.suez.edu.eg

## Figure Legend

In the original publication [1], there was a missing part in the legend for Figure 5. The correct legend appears below.

**Figure 5.** (**A**) Immunohistochemical staining of the cerebral cortex, CA region and dentate gyrus with Iba-1. Negative and non-observed immunostaining were seen in control, L-arginine (LA) and L-carnitine (LC) groups. Fipronil (FPN) group showed positive brownish Iba-1 immunoreactive microglia with numerous fine branching processes nuclei. Reduced immunoreactivity of microglia in FPN + LA and FPN + LC treated groups was seen [Anti-Iba-1 × 400]. (**B**) Immunoreactive parts percentage (IRP%) of Iba-1 protein expressed as mean ± SE. Symbols **, *** indicates significant *p* value < 0.01 and 0.001, respectively. The symbol a × b means a was statistically more varied than b; a × c means a was statistically more varied than c; and b × c means b was more statistically varied than c.

In addition, there was a mistake in the legend for Figure 6. The name of the immunohistochemical marker was mistyped as Iba-1; however, the correct one is DCX. The correct legend appears below.

**Figure 6.** (**A**) Immunohistochemical staining of the cerebral cortex, CA region and dentate gyrus with DCX. Weak perinuclear membrane reaction was seen in control, L-arginine (LA) and L-carnitine (LC) groups. The Fipronil (FPN) group showed intense positive brownish immunoreactive neurons in the subgranular and granular cell layers. Reduced immunoreactivity of microglia in FPN + LA and FPN + LC-treated groups was seen (Anti-DCX 400×). (**B**) Immunoreactive parts percentage (IRP%) of DCX protein expressed as mean ± SE. Symbol a × b means a was statistically more varied than b; a × c means a was statistically more varied than c; and b × c means b was statistically more varied than c. Symbols **, *** indicate significant *p* values < 0.01 and 0.001, respectively. 

## Error in Figure

In the original publication [1], there was a mistake in Figures 4 and 6 as published. Figure 4e was misdragged, and the dentate gyrus of the fipronil +L-carnitine group was misdragged in Figure 6 for DCX immunohistochemistry. The corrected Figure 4 and Figure 6 appear below.

The authors state that the scientific conclusions are unaffected. This correction was approved by the Academic Editor. The original publication has also been updated.

## Figures and Tables

**Figure 4:**
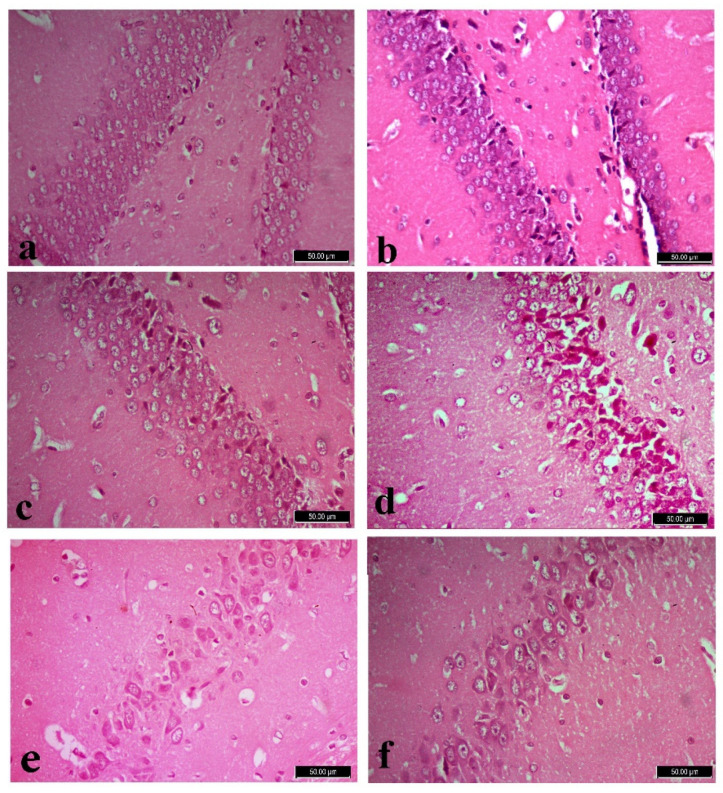
Dentate gyrus of control (**a**), L-arginine (**b**), L-carnitine (**c**) and fipronil (FPN)-treated rats (**d**). FPN-treated rat (**d**) showed shrunken, darkly stained granule cells with cytoplasmic vacuolations among few nearly normal granules cells with large vesicular nuclei. Improvements were observed in FPN + L-arginine and FPN + L-carnitine-treated groups (**e**,**f**). Stain: Hematoxylin and Eosin (H&E), magnification 400×.

**Figure 6:**
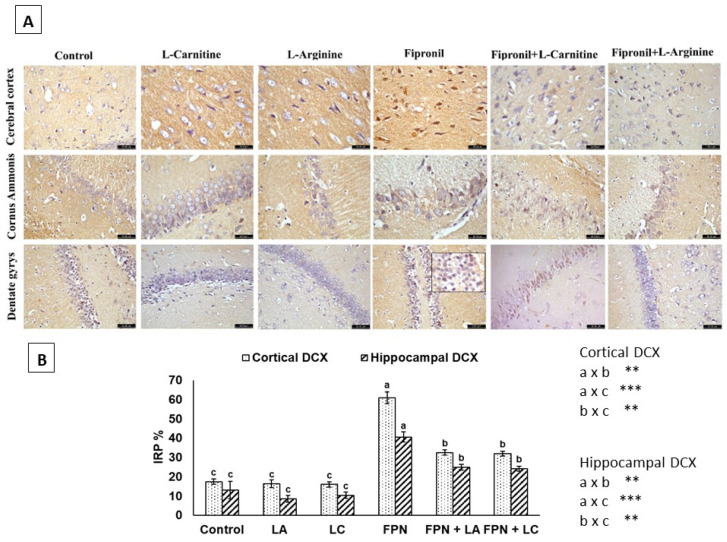
(**A**) Immunohistochemical staining of the cerebral cortex, CA region and dentate gyrus with DCX. Weak perinuclear membrane reaction was seen in control, L-arginine (LA) and L-carnitine (LC) groups. The Fipronil (FPN) group showed intense positive brownish immunoreactive neurons in the subgranular and granular cell layers. Reduced immunoreactivity of microglia in FPN + LA and FPN + LC-treated groups was seen (Anti-DCX 400×). (**B**) Immunoreactive parts percentage (IRP%) of DCX protein expressed as mean ± SE. Symbol a × b means a was statistically more varied than b; a × c means a was statistically more varied than c; and b × c means b was statistically more varied than c. Symbols **, *** indicate significant *p* values < 0.01 and 0.001, respectively.
